# Temperature Dependence of Strain-Induced Crystallization in Silica- and Carbon Black-Filled Natural Rubber Compounds

**DOI:** 10.3390/polym17162266

**Published:** 2025-08-21

**Authors:** Gaurav Gupta, André Wehmeier, Rene Sattler, Jens Kiesewetter, Mario Beiner

**Affiliations:** 1 Fraunhofer IMWS, Walter-Hülse-Str. 1, 06120 Halle (Saale), Germany; 2 Evonik Operations GmbH, Bruehler Str. 2, 50389 Wesseling, Germany; 3 Naturwissenschaftliche Fakultät II, Martin-Luther-Universität Halle-Wittenberg, 06099 Halle (Saale), Germany

**Keywords:** natural rubber, strain induced crystallization, nanofillers, tires

## Abstract

The results of strain-induced crystallization (SIC) studies on natural rubber compounds containing different amounts of carbon black and silica are reported. Two-dimensional wide-angle X-ray diffraction (2D WAXD) experiments were performed to quantify the degree of SIC at ambient and enlarged temperatures. The influence of temperature and filler system on the degree of crystallinity of natural rubber was investigated, since the estimated temperatures in truck tire treads are in the range 60–80 °C. Interestingly, the degree of crystallinity for silica-filled natural rubber compounds was commonly at least similar or higher compared to carbon black-filled compounds with identical filler mass fraction. In addition, it was demonstrated that the temperature dependence of natural rubber compounds containing silica and carbon black is also similar. In both cases the SIC disappeared slightly above 100 °C. Hence, it was concluded that the SIC behavior is most likely not the decisive factor for the different abrasion resistance of silica- and carbon black-reinforced natural rubber compounds for truck tire treads. This is an important insight considering the rising demand for sustainable rubber compounds for truck tire treads with low CO_2_ emissions as well as reduced abrasion.

## 1. Introduction

Silica/silane systems and their use in “Green Tire” technology for passenger car tire tread compounds are today state-of-the-art around the globe [1,2]. In particular, S-SBR/BR-based tread compounds, functionalized and/or extended with various plasticizers like resins [3,4] or liquid polymers [5,6] and/or blended with additional polymers like natural rubber [7] or synthetic isoprene rubber [8], are described in various publications. However, the application of primarily silica/silane-reinforced truck tire tread compounds based only on natural rubber (NR) has not widely penetrated the market.

Several reasons why silica/silane systems might not substitute for carbon black in such an application have been discussed at conferences and in the literature [9,10,11]. Even though laboratory indicators show the desired advantages regarding rolling resistance and wet grip indicators, the main disadvantage mentioned is a ten to twenty percent loss in abrasion resistance in full silica/silane systems compared to carbon black-reinforced truck tire tread compounds [12,13]. Similar disadvantages regarding abrasion resistance are also reported for natural rubber compounds filled with hybrid filler systems where carbon black is gradually replaced with large amounts of silica/silane at constant overall filler mass fraction [12,13,14]. Several reasons why this drawback might occur are discussed, e.g., higher mixing energy [15,16]; hindered silane/rubber coupling due to remaining proteins and amino acids [17,18], respectively; missing vinyl groups [19]; and others. An expected reason for the reduced abrasion resistance of earlier tested silica/silane-filled model compounds for truck tire treads might be a different influence of chemical-bonded silica/silane fillers compared to physically adsorbed carbon black on the strain-induced crystallization (SIC) of the natural rubber matrix.

In this paper, the results of investigations of SIC in natural rubber compounds are reported comparing different reinforcing systems, namely carbon black and silica/silane systems, at different filler loadings. Two-dimensional wide-angle X-ray diffraction (2D WAXD) measurements were performed to quantify the degree of SIC at ambient and heightened temperatures, since the typical temperature of a truck tire tread used on a long-haul truck is about 60–80 °C. We think that the results of this study can contribute to a better understanding of the reasons for differences in abrasion resistance between carbon black- and silica/silane-filled natural rubber compounds, which is required to develop efficient optimization strategies for the latter.

## 2. Materials and Methods

### 2.1. Materials

The matrix of the compound used for the investigations was pure natural rubber, a standard Malaysian rubber (SMR 10), masticated to a Mooney value of 60–70 Mooney units. The reinforcing systems were on the one hand the highly reinforcing, widely used carbon black N234, and on the other hand the highly dispersible precipitated silica ULTRASIL^®^ 7000 GR (Evonik Operations GmbH, Wesseling, Germany) in combination with the bi-functional silane Si 266^TM^ (Evonik Operations GmbH, Germany). Both active fillers were varied in their amounts as depicted in Table 1.

The vulcanization systems were adapted in order to achieve the same Shore-A-hardness for the standard carbon black compounds with 55 phr filler and the standard silica/silane compounds with 55 phr filler. This is a commonly applied criterion in the tire industry. The unit phr stands here for “parts per hundred rubber”, i.e., the relative mass content of other components in a recipe compared to the rubber content. In case of the system using di-sulfidic silane Si 266^TM^, additional sulfur compared to carbon black was needed to achieve the needed flexible long sulfur chain length for both polymer–polymer and silica–silane–polymer crosslinking.

### 2.2. Rubber Processing

The mixing was carried out in a 1.5 L intermeshing mixer using a three-stage mixing protocol. Within the first stage, the polymer and the reinforcing filler systems were mixed for 5 min at around 145 °C to ensure good hydrophobation of the silica by the bi-functional silane. The carbon black compounds were mixed in the same way so that no differences would occur due to different energy input or shearing of the compounds. The second stage was a three-minute re-mill step without any addition of further ingredients. It was also carried out at a batch temperature around 145 °C. The accelerator systems were added in a third stage and mixed at temperatures below 110 °C for 2 min. Measurements using a MonTech Dispertester 3000 (MonTech Werkstoffprüfmaschinen GmbH, Buchen, Germany) with 100 times magnification showed that the macro-dispersion of all compounds in this study was >97%, indicating very good filler dispersion.

### 2.3. Methods

Two-dimensional wide-angle X-ray diffraction (2D WAXD) patterns were recorded using a VANTEC500 area detector (Bruker AXS, Karlsruhe, Germany) with Ni-filtered Cu Ka radiation at a sample-detector distance of about 8.95 cm. The strain-dependent measurements were performed using a compact tensile stage enabling the uniaxial deformation of small shoulder bars with an initial length of the rectangular region of about 8 mm and a cross-section of 2 × 2 mm^2^ up to 400% strain. The true local strain close to the beam was measured using an optical method based on tracer points on the rubber sample.

Temperature-dependent measurements were performed on rubber stripes pre-strained to about 330% strain and then fixed in a frame mounted on a temperature stage integrated in the 2D WAXD chamber. The initially obtained strain was detected optically. To achieve good heat transfer between the heated block of the stage and the sample, a specially designed metal piece was used. All 2D WAXD measurements were performed under vacuum.

The areas of crystalline reflections A_cryst_ and the underlying amorphous halo A_am,total_ were determined from the azimuthally integrated 2D WAXD pattern. A linear baseline was subtracted from the measured WAXD pattern in the q range 0.8 to 2 Å^−1^ assuming that the shape of the amorphous halo was in principle only weakly strain-independent. Based on this approximation and the shape of the amorphous halo in the unstretched state, the total area of the amorphous contributions A_am,total_ was estimated in the case of stretched semi-crystalline NR compounds.

## 3. Results

**Figure 1:**
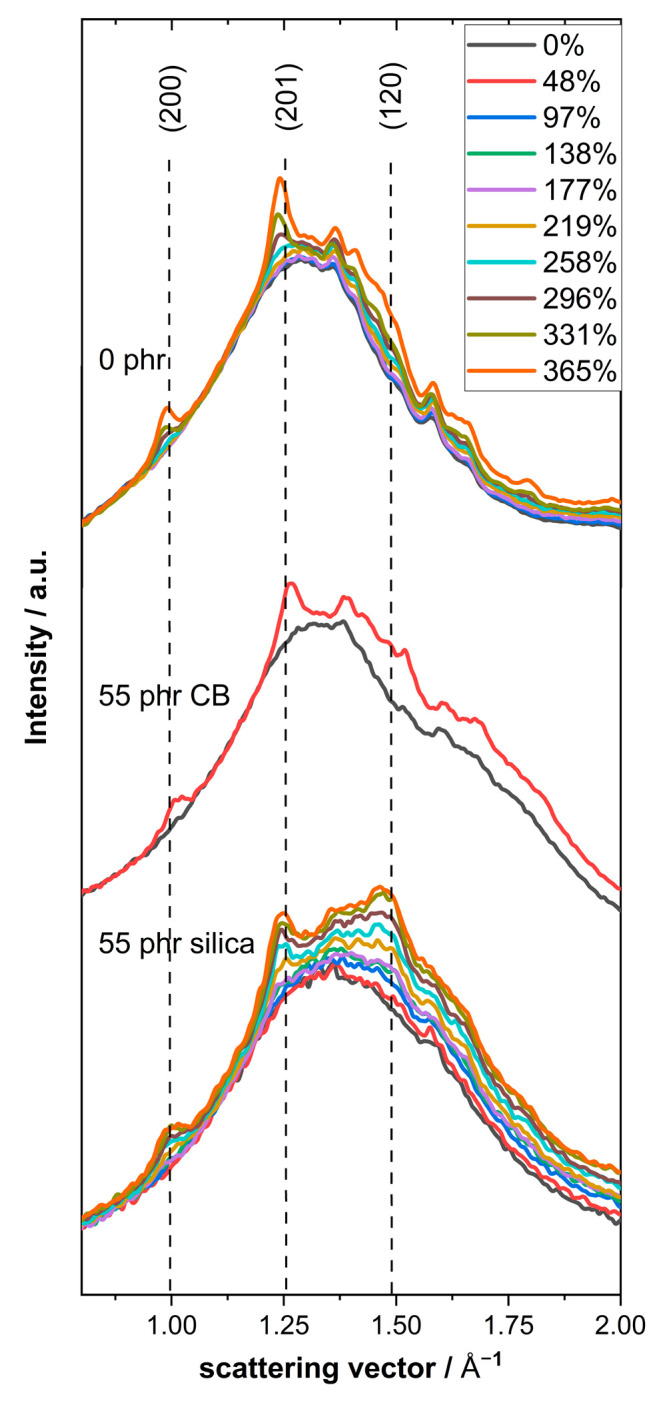
Curves for the scattering intensity I vs. scattering vector q for (**top**) natural rubber, (**middle**) a NR compound containing 55 phr carbon black, and (**bottom**) a NR compound containing 55 phr silica as obtained from an azimuthal integration of the 2D patterns of differently stretched samples.

The central aim of this work was to compare the strain-induced crystallization (SIC) in natural rubber compounds filled with 40 to 70 phr silica or carbon black. In the first step, 2D WAXD measurements were performed on samples under uniaxial deformation with strain values ranging from 0 to 400% at room temperature. The 2D WAXD patterns for vulcanized natural rubber (NR) and corresponding NR compounds filled with 55 phr silica and 55 phr carbon black are shown in Figure 1 as representative examples. Crystalline reflections appear above 200% strain, indicating the occurrence of SIC in natural rubber. The position of the stronger crystalline reflections is in good agreement with the q values expected for the (200), (201) and (120) lattice planes commonly found in crystalline natural rubber at about 1.0, 1.25 and 1.5 A^−1^, respectively [20,21,22]. The strongest crystalline reflections for the NR compounds filled with carbon black and silica appeared at quite similar positions.

Considering the pattern for undeformed samples in more detail, one can observe that the shape of the amorphous halo in unfilled NR and NR compounds filled with Ultrasil 7000 GR silica is seemingly similar, while that for NR compounds filled with N234 carbon black is significantly broader since an additional shoulder centered at higher q values (about 1.70 to 1.75 A^−1^) appears. The intensity of this shoulder significantly increases with increasing carbon black content, indicating that this feature is related to carbon black, while the weak shoulder appearing close to 2 Å^−1^ is obviously caused by additives used in commercial NR compounds (for details, see Supplementary Material, Figure S2b). Looking more carefully at the data for unfilled NR and silica-filled NR, one can also see that the peak width in the silica filled compounds is significantly enlarged, indicating extra contributions near 1.5 Å^−1^.

These observations are relevant if the degree of crystallinity D_C_ of the natural rubber matrix has to be estimated and compared for NR compounds containing different kinds of filler and different filler fractions. An important question for the calculation of D_C_ is then to what extent NR contributes to the area of the amorphous halo, and how to estimate this contribution, denoted A_am,NR_. An appropriate evaluation method has to be derived and applied. Accordingly, the equation

D_C_ = A_cryst_/(A_cryst_ + C_NR_ A_am,total_) (1)

is commonly used in this work to estimate the degree of crystallization D_C_ of the NR matrix, where A_cryst_ is the total area of the crystalline reflections belonging to NR, A_am,total_ is the experimentally determined area of the entire amorphous halo in the q range from 0.8 to 2 Å^−1^, and C_NR_ is a compound- and model-specific correction factor used to estimate A_am,NR_ = C_NR_ A_am,total_, as discussed in more detail below. The determination of D_C_ is a complicated issue, since different aspects will influence the overall scattering pattern in cases of NR compounds undergoing SIC. In particular, the contribution of NR to the amorphous halo is hard to determine. Different approaches can be considered in cases of fillers with quite different scattering behavior. Two reasonable models giving somewhat limiting values for C_NR_ are discussed in the next two paragraphs.

In order to learn more about filler-related contributions to the amorphous halo, a quantitative analysis of the scattering patterns for all undeformed NR compounds based on a fit with three Lorentzian functions was performed (cf. Supplementary Material, Figure S1 and Table S1). Further analysis of the area of the Lorentzian function with a peak maximum at intermediate q values (1.57 Å^−1^ for silica-filled and 1.73 Å^−1^ for CB-filled NR compounds) shows that this contribution corresponds to the filler fraction in both cases, while the other two peaks in the fit (at about 1.33 Å^−1^ and 2.03 Å^−1^) are related to non-filler contributions to the amorphous halo, i.e., NR and all other components in the compounds. This is confirmed by (i) the position of the filler-related peaks (cf. Supplementary Material, Figure S2 and Table S1) and (ii) the dependence of the area of this peak on filler content. It is observed that the area of the intermediate peaks is approximately proportional to the filler mass fraction for both series of NR compounds (cf. Supplementary Material, Figure S3). The relative contributions related to the non-filler components decrease accordingly with increasing filler fraction. Hence, the NR contributions to the area of the amorphous halo should be, in good approximation, proportional to the mass fraction of NR in compounds containing fillers. Based on these findings as reported in the Supplementary Material, the NR mass fraction correction C_NR,MC_ = m_NR_/m_total_ was used to calculate the degree of crystallinity D_C,MC_ of NR in compounds containing different amounts of filler and additional additives as one approximation.

An alternative approach considers the fact that the absorption of silica is obviously much stronger compared to those of NR, additives and carbon black. This is confirmed by a strong reduction in the total area of the amorphous halo with increasing filler content in silica-filled NR compounds (cf. Supplementary Material, Figure S4) if measured under otherwise identical conditions. Using an extreme model approach, one can assume that there are basically no contributions of silica to the amorphous halo, since absorption and multiple scattering prevent scattered photons from leaving the silica particles/clusters and therefore the entire sample. Considering this assumption, an absorption-based correction C_NR,AC_ = m_NR_/m_non-filler_ = 0.826 (where m_non-filler_ includes only the non-filler components) could be used to calculate the degree of crystallinity D_C,AC_ of the NR fraction in silica-filled compounds. For carbon black-filled NR compounds, the mass fraction correction C_NR,MC_ can seemingly be maintained despite absorption effects, since there is basically no dependence of the total area of the amorphous halo on the filler content under otherwise identical measurement conditions (cf. Supplementary Material, Figure S4).

Both approaches were applied to silica-filled NR compounds in order to demonstrate the consequences of the considered assumptions/models. Note that it remains extremely complicated to address this problem experimentally since (i) there are many influencing factors and components in NR compounds as well as (ii) no alternative experimental methods like classical DSC that could give the usual absolute values for the degree of crystallinity D_C_. Such standard methods are unavailable in cases of NR undergoing SIC.

**Figure 2:**
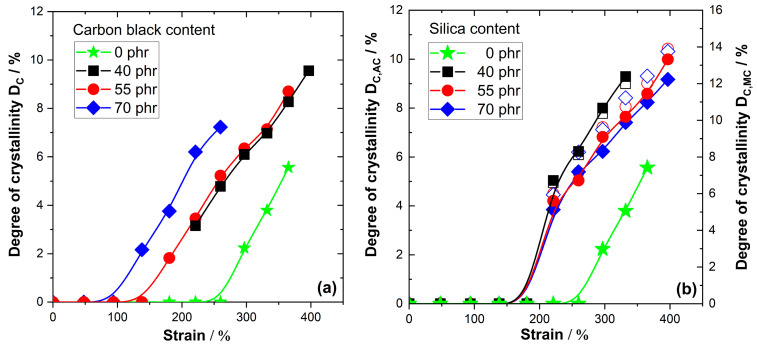
Degree of crystallinity depending on strain calculated from 2D patterns for various natural rubber compounds containing (**a**) different amounts of carbon black and (**b**) different amounts of silica with 6.6 phr silane. Filled symbols in part (**b**) are calculated from C_NR,AC_ in Table 1 and plotted on the left y-axis; open symbols in part (**b**) are calculated based on the C_NR,MC_ values and plotted on the right y-axis. The color encodes the filler contents as given in the legends.

The strain-dependent D_C_ values for NR compounds filled with different amounts of silica and carbon black are compiled in Figure 2. An amplification of SIC compared to unfilled NR with similar crosslinking density is observed for both types of fillers—silica and carbon black. For carbon black-filled NR compounds only the mass-based correction factor C_NR,MC_ is applied to calculate D_C_. The D_C_ values for silica-filled NR compounds are, interestingly, similar to or even higher than those for carbon black-filled compounds, depending on the model used to estimate D_C_. The absorption correction for silica-filled compounds gives D_C,AC_ values similar to the D_C_ values calculated for carbon black-filled compounds, while the NR mass fraction correction interestingly gives much higher D_C,MC_ values for the silica-filled NR compounds. Such a significant increase in D_C_ seems to be a bit unlikely, although the covalent bonds between silica filler and NR matrix via silane coupling agents may improve the situation since NR chains cannot easily be detached from the filler at high strain.

On the other side, there are also clear differences between carbon black- and silica filled-compounds. In the case of carbon black-filled compounds, the D_C_ values for the NR matrix increase nearly linearly with the applied strain above the onset of SIC, as observed for unfilled NR. The extrapolated onset of SIC significantly shifts to lower strain values with increasing filler content. The onset appears at about 250% strain for unfilled NR, while it is in the range between 150% and 100% strain for the NR compounds filled with 40 to 70 phr N234 carbon black. D_C_ values of about 9–10% are achieved for NR compounds filled with 40 phr and 55 phr carbon black at 400% strain compared to D_C_~6% for unfilled NR. The situation in NR compounds containing 40 to 70 phr silica is slightly different. In this case, the differences between NR compounds containing different amounts of silica are quite small. Moreover, the onset of SIC commonly appears in silica filled compounds at a strain of about 180% and the D_C_ values rise more abruptly shortly after the onset before a nearly linear increase is achieved. The absorption-corrected D_C,AC_ values are in the range 9–10% before the sample breaks for all investigated silica contents. The D_C,MC_ values from NR mass fraction correction are much higher, in the range of about 14%.

**Figure 3:**
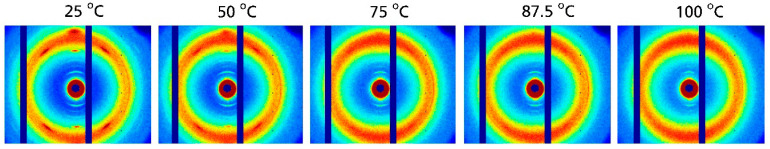
2D WAXD pattern for unfilled natural rubber vulcanized with 2 phr sulphur measured at 330% strain and different temperatures.

In the second step, the temperature dependence of SIC at a fixed strain of about 330% was investigated. The 2D scattering patterns observed for unfilled NR at different temperatures are presented in Figure 3. Crystalline reflections similar to those obtained in strain-dependent measurement are clearly seen at room temperature. Prominent (200) and (120) reflections are found along the vertical direction, i.e., perpendicular to the applied uniaxial deformation, while four peaks are observed for the (201) planes, which are tilted relative to the vertical direction in the 2D pattern. This is the expected behavior and in line with findings that are commonly reported in the literature [20,21,22]. All crystalline reflections disappear systematically with increasing temperature. At 100 °C the crystalline reflections are basically absent, and only weak indications for a tiny crystalline fraction that remains at this temperature are observed.

**Figure 4:**
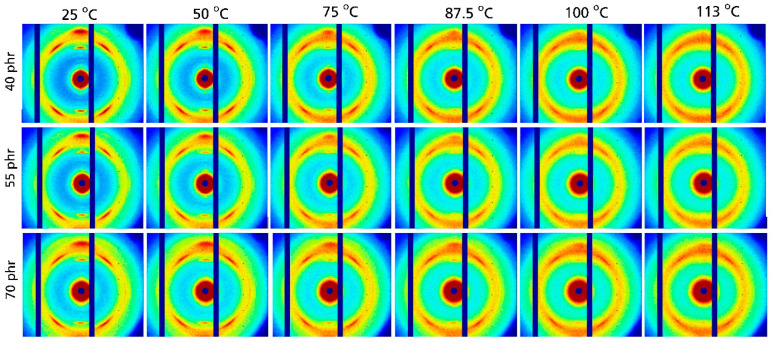
2D WAXD patterns for natural rubber compounds filled with different amounts of carbon black measured at 330% strain and different temperatures. The labels on the left hand side indicate the filler content.

**Figure 5:**
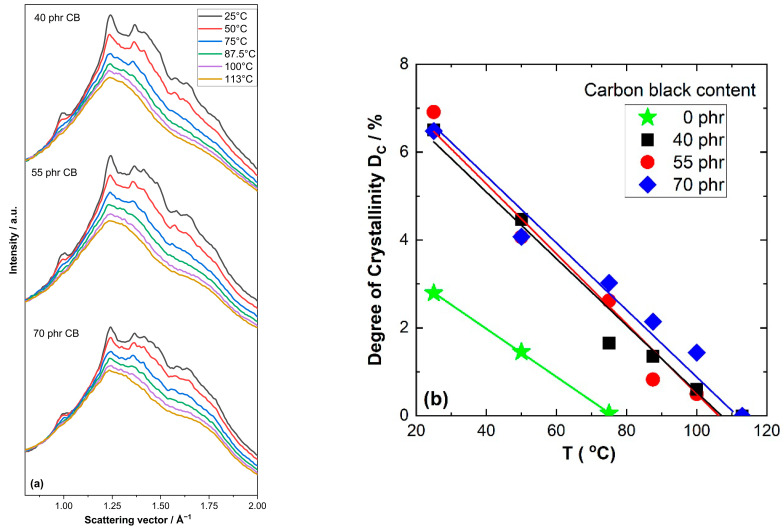
(**a**) Scattering intensity vs. scattering vector as function of temperature for natural rubber compounds containing different amounts of carbon black. (**b**) Degree of crystallinity D_C_ vs. temperature for investigated NR compounds containing carbon black.

Very similar effects are seen in the 2D WAXD patterns for NR compounds containing different amounts of carbon black presented in Figure 4. Also in this case, the characteristic reflections of crystalline NR are found at 25 °C together with a systematic decrease in their intensity at higher temperatures. The intensity of the crystalline reflections at 100 °C is seemingly a bit higher compared to that in unfilled samples. Hence, the 2D pattern was also recorded at 113 °C, resulting in a situation similar to that observed for unfilled NR at 100 °C. A visual inspection of the 2D WAXD pattern indicated that the crystalline fraction is still present above 100 °C, i.e., it disappears at slightly higher temperatures in carbon black-filled NR compounds compared to unfilled NR. A more detailed analysis of the temperature dependence of D_C_ was made based on azimuthal integration of the 2D patterns shown in Figure 4. The resulting WAXD patterns are given in Figure 5a. All relevant reflections for crystalline NR are observed. A systematic reduction in the intensity of the crystalline reflections with increasing temperature is seen for all investigated carbon black contents. A further analysis focusing on the temperature dependence of the degree of crystallization shows a nearly linear decrease in D_C_ with increasing temperature (Figure 5b). This trend is observed for all investigated filler contents ranging from 40 to 70 phr carbon black. A linear extrapolation shows that the crystalline fraction disappears at about 105 to 115 °C, with a somewhat higher temperature observed for 70 phr carbon black. However, in general, the degree of crystallinity of the rubber matrix is weakly affected by carbon black content in the range 40 to 70 phr.

The situation for unfilled NR is characterized by smaller D_C_ values at 25 °C, as also obtained in strain-dependent measurements, as well as a crystalline fraction that disappears at significantly lower temperatures (about 80 °C) compared to carbon black-filled NR compounds. Note that the absolute D_C_ values at 25 °C are commonly a bit lower compared to the related values from strain-dependent measurement reported above. This is possibly due to stress relaxation effects that may appear under slightly different conditions in the case of temperature-dependent measurement performed at a fixed strain of about 330%. Significant differences in the local strain are unlikely, since the situation was investigated by an optical method, although a certain deviation of the local strain from 330% cannot be absolutely excluded. The linear temperature dependence of D_C_ is unaffected by these minor experimental uncertainties.

**Figure 6:**
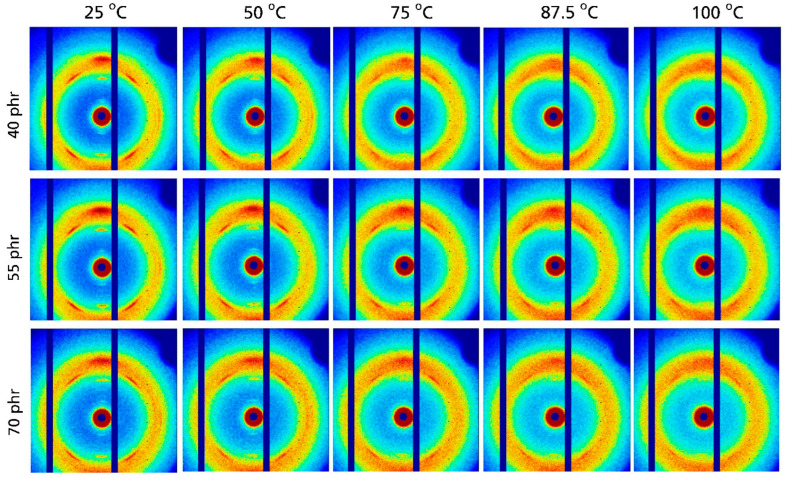
2D WAXD patterns for natural rubber compounds filled with different amounts of silica measured at 330% strain and different temperatures. The filler content is labeled on the left.

**Figure 7:**
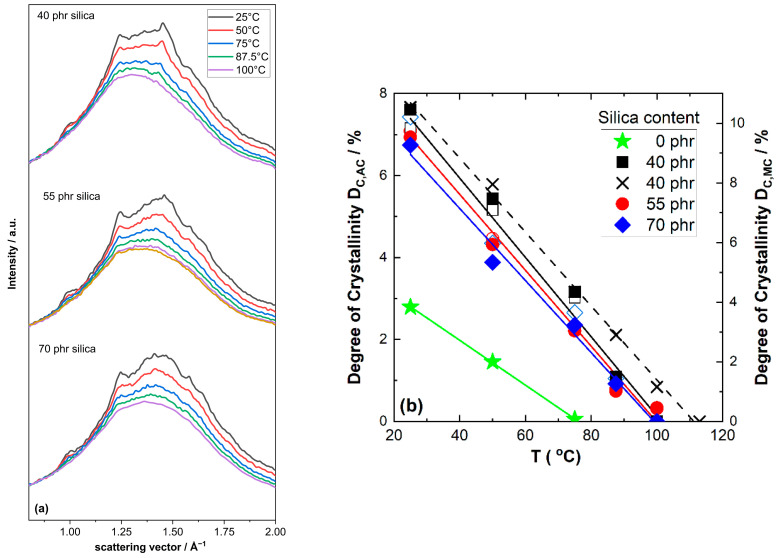
(**a**) Scattering intensity vs. scattering vector as function of temperature for natural rubber compounds containing different amounts of silica. (**b**) Degree of crystallinity vs. temperature for investigated compounds containing silica. Filled symbols are calculated based on C_NR,AC_ in Table 1 and plotted on the left y-axis, open symbols are calculated based on the C_NR,MC_ values in Table 1 and plotted on the right y-axis.

The 2D WAXD patterns for NR compounds containing 40 to 70 phr silica are compiled in Figure 6. As in other NR compounds, significant reflections indicating the presence of crystalline NR are observed at 25 °C. The intensity of these reflections in these compounds also decreases with increasing temperature. At 100 °C there is basically only a non-oriented amorphous halo remaining. Clear indications of a crystalline fraction are absent in this temperature range for all investigated silica contents. This observation is also confirmed by the WAXD patterns obtained from azimuthal integration of the 2D patterns shown in Figure 7a. With increasing temperature, these patterns show a systematic intensity reduction for the (200), (201) and (120) reflections appearing at about 1.0, 1.25 and 1.5 Å^−1^, respectively. At 100 °C the intensity of the crystalline reflections is quite small and practically approaches zero. This basic impression is also confirmed by a further analysis of the WAXD patterns focusing on the degree of crystallinity. As shown in Figure 7b, the D_C_ values for NR compounds containing silica also decrease nearly linearly with temperature. The extrapolated temperatures where the crystalline NR fraction disappears are only a few Kelvin lower compared to those observed for NR compounds filled with carbon black: independent of the filler content, a temperature of about 102 °C ± 2 °C is observed for NR compounds containing 40 and 70 phr silica; only the temperature for the middle batch filled with 55 phr silica is slightly higher (about 110 °C). The slight scatter in the data for NR compounds with different silica contents possibly reflects experimental uncertainties. The crystalline NR fraction also disappears for these compounds at higher temperatures compared to unfilled NR, where this happens at about 75–80 °C, i.e., significantly below 100 °C. Note that the obtained D_C_ values again depend significantly on the correction method applied. The absorption-corrected values D_C,AC_ are comparable to the D_C_ values obtained for carbon black-filled NR compounds in Figure 5, while the NR mass-corrected values D_C,MC_ for silica-filled NR compounds are significantly higher (nearly 50%).

Figure 7 also shows that there is a certain scatter in the D_C_ values for batches with an identical recipe prepared under identical processing conditions. This scatter is small but comparable to the differences obtained for batches containing different amounts of silica. To what extent this might be due to batch-to-batch variations, local inhomogeneities in highly filled NR compounds, or experimental uncertainties of the used scattering techniques and data evaluation methods requires further investigation. The main findings of this study are, however, unaffected by these remaining uncertainties.

In summary, one can conclude from the observations in this study that:

(i) Strain-induced crystallization (SIC) of NR is significantly improved by the presence of 40 to 70 phr of nanofillers like carbon black or silica. The onset of SIC appears at lower strain values, and D_C_ values are commonly higher if nanofillers are incorporated. The D_C_ values for silica-filled NR compounds are at least similar to or higher than those of carbon black-filled compounds with similar filler mass fractions, depending on the correction factors applied (absorption correction or mass correction). The onset of strain-induced crystallization commonly appears at lower macroscopic strain values for filler-containing NR compounds compared to unfilled NR and the actual D_C_ values for a given strain above the onset are higher for filled NR compounds. Certain differences are indicated between carbon black- and-silica filled NR compounds. This is especially seen close to the onset of strain-induced crystallization. The onset commonly appears at close to 180% for silica-filled compounds, while a slight shift with filler content is indicated in the case of carbon black-filled compounds. In addition, the rise directly after onset appears more abruptly in the case of silica-filled samples than in carbon black-filled samples, where the rise with increasing strain is nearly linear.
(ii) A linear decrease in the degree of crystallinity Dc at 330% strain is observed with increasing temperature for various NR compounds filled with 40 to 70 phr silica or carbon black. The extrapolated temperatures where the crystalline NR fraction disappears range from 100 to 115 °C for NR compounds filled with silica or carbon black, respectively. This is significantly higher compared to the related temperature estimated for unfilled NR, which is about 80 °C. The data indicate that the disappearance of the crystalline NR fraction occurs at temperatures a few Kelvin higher for carbon black compounds as compared to silica compounds containing a similar mass fraction of filler.

## 4. Discussion

Considering the commonly expected relevance of strain-induced crystallization effects for the abrasion resistance of NR-based truck tire treads, it is important to understand the influence of different nanofillers on this phenomenon. In particular, this is interesting in the light of ongoing activities towards truck tires with reduced rolling resistance, where carbon black is replaced by silica fillers in tread compounds.

The results of our study indicate that there are no strong qualitative differences in SIC between carbon black- and silica-filled NR compounds. In both cases an improvement in SIC is achieved by the presence of nanofillers, i.e., the degree of crystallization D_C_ of filled NR compounds is at a given strain significantly higher compared to unfilled NR. This is understandable considering the fact that the local strain in a highly filled rubber compound containing a filler network is locally much higher compared to the macroscopically applied strain. This results in higher D_C_ values for highly-filled NR compounds and can also explain why the onset of crystallization appears at lower strains in filled NR compounds. In such compounds, even at low macroscopic strains, the critical deformation needed to crystallize the NR matrix can be reached locally. In this respect, there are no qualitative differences regarding SIC in CB- and silica-filled NR compounds. Interestingly, the D_C_ values observed for silica-filled NR compounds at high strain (>300%) and room temperature are at least similar (absorption-based correction) or even higher (NR mass fraction correction) than those of comparable carbon black-filled NR compounds. Considering further details of the SIC behavior, there are obviously also certain differences between NR compounds reinforced with carbon black and silica. These differences might be minor, but they are seemingly significant. There are at least three features that should be mentioned here: (1) the onset of SIC shifts with filler content to lower strains in CB but is nearly filler fraction-independent in silica-filled NR compounds at ambient temperature, (2) the increase in D_C_ shortly after onset occurs more abruptly with silica fillers, and (3) the disappearance of the crystalline NR fraction at a given strain occurs at slightly higher (about 5 K) temperatures for CB-filled NR compounds compared to those containing silica.

**Figure 8:**
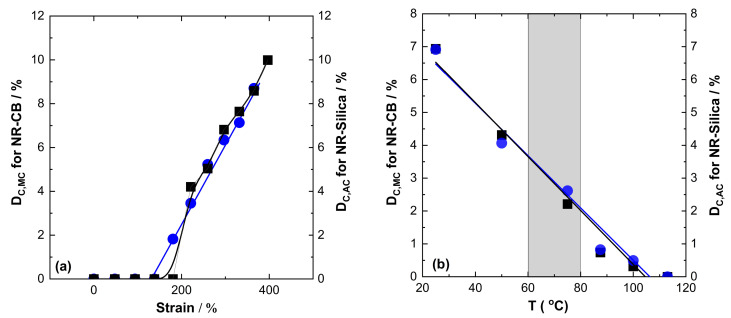
D_C_ values quantifying SIC behavior (**a**) at 25 °C depending on applied uniaxial strain and (**b**) at 330% strain but different temperatures for natural rubber compounds containing 55 phr silica (squares) and 55 phr carbon black (circles). D_C,MC_ values are given for carbon black-filled NR (circles, left axis) and D_C,AC_ values are shown for silica-filled NR (squares, right axis). The temperature range most relevant for truck tire treads is indicated by the grey shadowed area.

If we focus on the most application-relevant conditions and filler contents, there are obviously no serious disadvantages of silica-filled NR compounds compared to carbon black-filled NR compounds containing up to 70 phr filler. The D_C_ values of the NR matrix at ambient temperature and in the temperature range relevant for truck tire treads (60–80 °C) of the silica compound are at least comparable to those of NR compounds containing carbon black under these conditions. This is clearly visible in Figure 8, where the situation in NR compounds filled with 55 phr silica or carbon black is compared in detail. This comparison demonstrates that the strain dependence D_C_ of the silica-filled compound is qualitatively comparable to that in the corresponding NR compound containing carbon black apart from slight differences close to the onset (Figure 8a), and that small differences in the temperature dependence of the D_C_ values should not cause disadvantages (Figure 8b). Only for the highest filler contents under investigation (70 phr) might there be indications of certain advantages of CB-filled NR compounds regarding SIC for low strain, since the onset of SIC already occurs at 100% strain close to environmental temperatures, and at high temperatures, where SIC survives at probably 5–10 K higher temperatures compared to silica-filled NR compounds containing the same mass fraction of filler. However, even at such high CB fractions—which are not commonly used in truck tire treads—there are indeed no significant regions in the relevant strain–temperature window where carbon black should impart real advantages compared to silica regarding the SIC of the natural rubber matrix in NR compounds. 

Hence, it can finally be concluded that the SIC behavior is most likely not the decisive factor for the different abrasion resistance of silica- and carbon black-reinforced NR compounds for truck tire treads. Whether minor differences in SIC achieved only under special conditions might have an influence on the abrasion of tires with silica-filled tread compounds, or other influencing factors are more relevant, cannot be determined here since tire abrasion remains a highly complex and hardly predictable phenomenon. From a practical point of view, however, it remains an extremely important task for materials science and of increasing application relevance to achieve a good compromise allowing the production of truck tires combining low CO_2_ emissions and reduced abrasion. Both aspects are extremely relevant for the sustainability of future cars.

## Figures and Tables

**Table 1:** Rubber formulations used.

	Unfilled	Silica-Filled			Carbon Black-Filled		
	Phr	Phr	Phr	Phr	Phr	Phr	Phr
**First stage**							
SMR 10	100	100	100	100	100	100	100
N234					40	55	70
Ultrasil 7000 GR		40	55	70			
Silane Si 266		6.6	6.6	6.6			
ZnO	3	3	3	3	3	3	3
Stearic acid	3	3	3	3	3	3	3
TMQ ^a^	1	1	1	1	1	1	1
6PPD ^b^	1	1	1	1	1	1	1
Wax	1	1	1	1	1	1	1
**Second stage**							
batch first stage							
**Third stage**							
batch second stage							
80% DPG ^c^		2.5	2.5	2.5			
Sulfur	1.4	2	2	2	1.4	1.4	1.4
CBS ^d^	1.6	1	1	1	1.6	1.6	1.6
Total phr	112	161.1	176.1	191.1	152	167	182
C_NR,MC_	0.893	0.621	0.568	0.523	0.658	0.599	0.549
C_NR,AC_	0.893	0.826	0.826	0.826	-	-	-

^a^ 2,2,4-tri-methyl-1,2-dihydrochinoline; ^b^ N-(1,3-dimethylbutyl)-N′-phenyl-p-phenylendiamine; ^c^ N,N′-*diphenylguanidine*; ^d^ N-cyclohexyl-2-benzo thiazolesulfenamide.

## Data Availability

The relevant data are provided in the paper.

## Supplementary Materials

The following supporting information can be downloaded at: https://www.mdpi.com/article/10.3390/polym17162266/s1. Details of the data evaluation methods used to analyze the WAXD results are provided as pdf document. Equation (S1); Table S1: Fit parameters from an approximation of a normalized WAXD pattern based on Equation (S1); Figure S1: Fitting of background-corrected WAXD patterns for NR compounds containing (a) 40 phr silica and (b) 40 phr carbon black based on Equation (S1); Figure S2: WAXD pattern for (a) carbon black and silica in the form of a controlled porous glass plate (2 mm thick, pore diameter 55 nm, porosity 55%) as well as (b) unfilled NR compound and a peroxidically crosslinked NR sample; Figure S3: Relative filler contribution of the filler-related peak to the area of the amorphous halo A_2_/A_total_ for undeformed NR compounds with different CB (circles) and silica (squares) contents quantified by filler mass fraction m_filler_/m_total_; Figure S4: Total area A_total_ of the amorphous halo in the range 0.8–2.5 Å^−1^ depending on filler mass fraction for carbon black- and silica-filled NR compounds.

## Abbreviations

The following abbreviations are used in this manuscript:

NR	Natural rubber
phr	Parts per hundred rubber
SCI	Strain-induced crystallization
WAXD	Wide-angle X-ray diffraction
