# Supplementary material for: Temperature Dependence of Strain-Induced Crystallization in Silica- and Carbon Black-Filled Natural Rubber Compounds
Source: Polymers (Basel). 2025 Aug 21;17(16):2266. doi: 10.3390/polym17162266 (PMC12390267; doi:10.3390/polym17162266)
Supplement: Supplementary file 1 [file polymers-17-02266-s001.zip › polymers-3758081-supplementary.pdf]

Supplementary Material

Temperature Dependence of Strain-Induced Crystallization in Silica- and Carbon Black-Filled Natural Rubber Compounds

Gaurav Gupta [1], Andre Wehmeier [2], Rene Sattler [1,3], Jens Kiesewetter [2] & Mario Beiner [1,3]

[1] *Fraunhofer IMWS, Walter-Hülse-Str. 1, 06120 Halle (Saale), Germany*
[2] *Evonik Operations GmbH, Bruehler Str. 2, 50389 Wesseling, Germany*
[3] *Martin-Luther-Universität Halle-Wittenberg, Naturwissenschaftliche Fakultät II, 06099 Halle (Saale), Germany*

Different contributions to the amorphous halo of NR compounds in the undeformed state

Azimuthally integrated 2D-scattering intensity data as function of scattering vector q of undeformed specimen for all investigated rubber compounds are analyzed using a fit with multiple Lorentzian functions. After background correction with a straight line fitting is done using

$$I(q) = \Sigma_n \; 2A_n w_n/(\pi \; (4(q-q_{max,n})^2 + w_n^2)) \qquad \text{(S1)}$$

with A_n being the peak area, $q_{n,max}$ the peak maximum position and w_n is the full width at half maximum height of the nth Lorentzian peak and I_0 an individual baseline correction. Three Lorentzian functions (n=3) are used for all filled NR compounds and two Lorentzian functions (n=2) are applied for the unfilled NR sample.

Representative fits for one carbon-black and one silica filled NR compound are presented in Figure S1. One can clearly see in both cases a main contribution with a maximum near $q_{1,max}$ = 1.32 Å^{-1} close to the position of the amorphous halo of unfilled NR. The peaks centered at intermediate values $q_{2,max}$ are mainly caused by the filler and appear at slightly different positions for silica and CB filed NR compounds. The observed $q_{2,max}$ values correspond approximately to those found for the amorphous halo of pure silica and carbon black being 1.51 A^{-1} and 1.69 Å^{-1}, respectively (Figure S2a). The weak peaks at large values $q_{3,max}$ are most likely related to a large extent to the additives being part of the rubber matrix as listed in Table 1. This can be concluded from Figure 2b showing that the shoulder in the original WAXD data which is responsible for this third peak is seen for all NR compounds studied here is absent in NR samples crosslinked with peroxide without further additives. All relevant fit parameters corresponding to an approximation based on Equation (S1) are summarized in Table S1.

Table S1. Fit parameters from an approximation of normalized WAXD pattern based on Equation (S1).

	Unfilled	Silica filled			Carbon black filled		
	0 Phr	40 phr	55 phr	70 phr	40 phr	55 phr	70 phr
A_1	8.060	6.462	5.930	5.669	6.986	6.619	6.338
A_2	0	2.174	2.747	3.062	1.479	1.885	2.279
A_3	0.669	0.473	0.385	0.294	0.389	0.306	0.194
A_{total}	8.729	9.109	9.062	9.025	8.854	8.810	8.811
$q_{1,max}$/ Å^{-1}	1.3136	1.3288	1.3426	1.3419	1.3255	1.3294	1.3321
$q_{2,max}$/ Å^{-1}	–	1.5755	1.5853	1.5840	1.7270	1.7304	1.7285
$q_{3,max}$ / Å^{-1}	2.0103	2.0082	2.0535	2.0216	2.0344	2.0388	2.0100

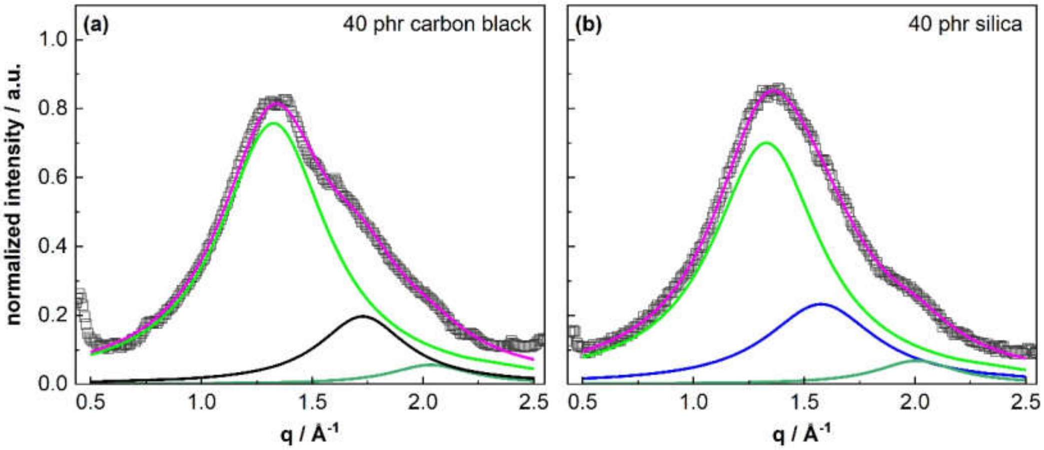

Figure S1. Fitting of background corrected WAXD pattern for NR compounds containing (a) 40 phr silica and (b) 40 phr carbon black based on Equation S1. The bold line (magenta) indicates the results of the fit. The three Lorentzian functions are indicated by additional thin colored lines (component 1-light green, component 2 – blue/black, component 3 – dark green).

A further analysis of the filler-related contributions A_2 to the entire amorphous halo as function of the filler mass fraction $C_{filler} = m_{filler}/m_{total}$ is shown in Figure S3. One can see that A_2 is nearly proportional the filler mass fraction C_{filler} for silica filled NR compounds. This tendency applies to also to carbon black filled NR compounds although certain deviations do exist in this case. The values of A_2 are generally lower than expected based on simple mass correction for carbon black filled NR compounds. These deviations might be due to a more complex overlap of the different contributions of the amorphous halo. Hence, it remains open whether or not this is a real effect, or an artefact of the complex fitting procedure applied here. In general, it is extremely complicated to separate different contributions to the amorphous halo in NR compounds containing more than ten individual components (cf. Table 1) since it is nearly impossible to deconvolute ten broad peaks with quite different intensities but similar peak maximum position superimposed in a WAXD pattern. This remains an

extremely challenging task even if the individual pattern of all components are available since multicomponent effects and scatter in the data will be always limiting factors.

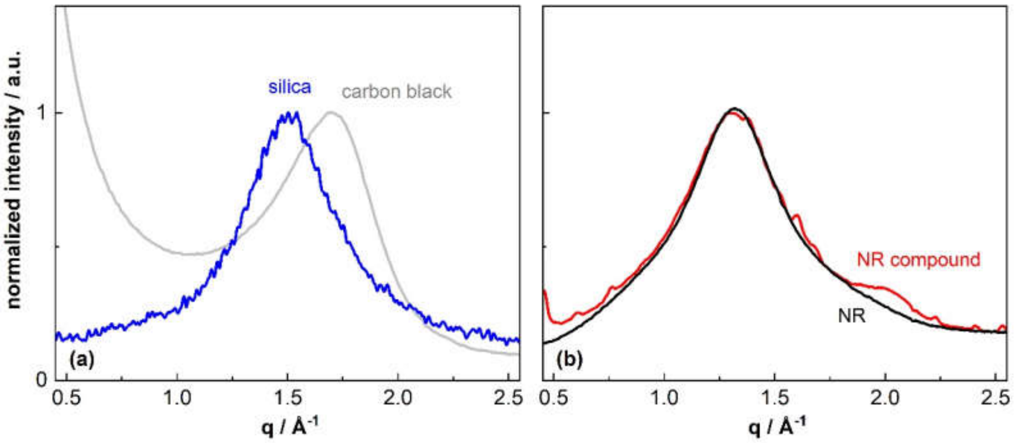

Figure S2. WAXD pattern for (a) carbon black and silica in form of a controlled porous glass plate (2 mm thick, pore diameter 55 nm, porosity 55 %) as well as (b) unfilled NR compound and a peroxidically crosslinked NR sample.

The final conclusion of this analysis of the amorphous halo is that **a simple mass correction of the different contributions might be a reasonable approximation allowing to estimate the area caused by the different components in filled NR compounds.** According to this approximation, the contribution of the crystallizable NR fraction to the amorphous halo can be estimated from its total area based on $A_{NR} = C_{NR,MC}\ A_{total} = m_{NR}/m_{total}\ A_{total}$ with $C_{NR,MC} = m_{NR}/m_{total}$ being a mass-based correction factor. Since only the NR contribution A_{NR} to the entire amorphous halo is relevant for the calculation of the degree of strain induced crystallization, D_c of NR in compounds containing filler and additives the NR mass correction factor $C_{NR,MC}$ is applied in Equation (1) to data from WAXD for all NR compounds of this work. There are definitively remaining uncertainties of this experiment-based approach, but other approaches may have even larger uncertainties since scattering cross sections, absorption factors and multiple scattering of all components will influence the overall scattering intensity in parallel.

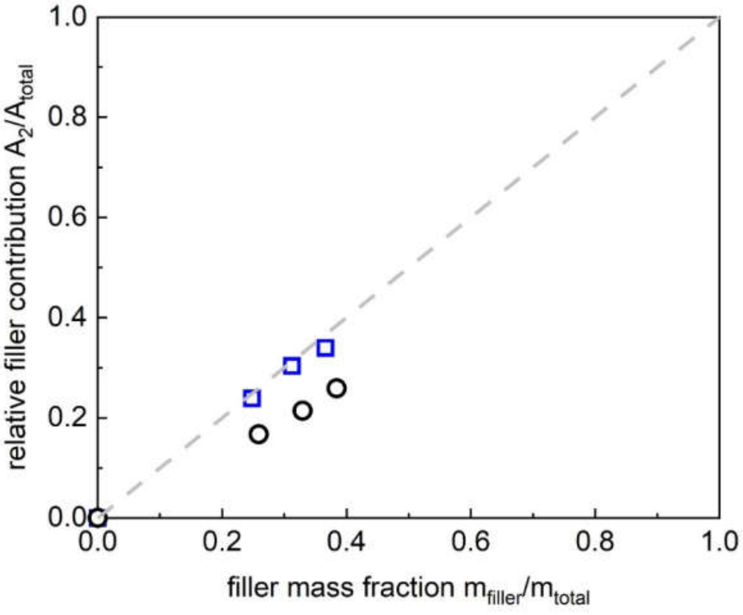

Figure S3. Relative filler contribution of the filler-related peak to the area of the amorphous halo A_2/A_{total} for undeformed NR compounds with different carbon black (circles) and silica (squares) contents quantified by the filler mass fraction m_{filler}/m_{total}. The dashed line indicates the assumed proportionality between both quantities.

Absorption related effects in differently filled NR compounds

A second aspect influencing the scattering pattern and the contribution of the fillers to the amorphous halo is their absorption behavior in NR compounds. In this regard there are significant differences between carbon black and silica. Since all investigated specimen have an equal thickness of about 2 mm and have been measured under practically identical conditions (constant beam intensity, sample-detector distance etc) the observed scattering intensities should give a suitable impression about the situation in differently filled NR compounds. In order to demonstrate the dependence on filler content and filler type the total area of the amorphous halo is calculated for all NR compounds. The results of this integration (after linear background correction) are given in Figure S4 and show clearly that silica filler causes a dramatic decrease in the integrated scattering intensity A_{total} while the addition of carbon black does not change A_{total} significantly. This justifies that silica filler result in a strong reduction of the X-ray scattering intensity while carbon black is not very relevant in this regard. This can be understood based on the assumption that the absorption within the silica particles/clusters is quite high. Based on that one may consider that the contribution of silica to the amorphous halo of NR compounds containing silica is extremely small (close to zero, i.e. $A_{filler} = 0$) while carbon black equally contributes to the amorphous halo. This would result in correction factors C_{NR} which should be differently calculated for silica and carbon black since $A_{total} = A_{NR} + A_{filler} + A_{add}$ would contain a carbon black contribution $A_{filler} = A_{CB} = m_{CB}/m_{total}$ proportional to its mass fraction while A_{total} for silica-filled compounds would not include a filler contribution $A_{filler} = A_{silica} = 0$ due to strong absorption of the scattered photons

inside the silica particles/clusters. Hence, the absorption-based correction factor $C_{NR,AC} = m_{NR} / (m_{NR} + m_{add})$ is an alternative approach for silica-filled NR compounds, which considers only the masses of the non-filler components. This results for the silica-filled NR compounds studied in this work in a fixed correction factor $C_{NR,AC} = 0.826$. Note that his is an extreme assumption which should give something like a limiting approximation considering the influence of absorption / multiple scattering on the contribution of the filler to the amorphous halo. The true value for the correction factor C_{NR} for silica might be in the range between the two extremes considered in this SI.

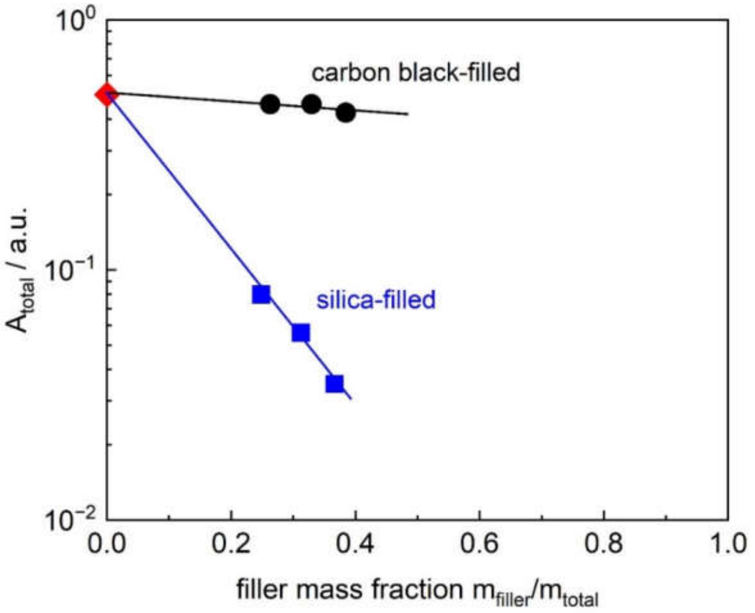

Figure S4. Total area A_{total} of the amorphous halo in the range 0.8 to 2.5 Å^{-1} depending on filler mass fraction for carbon black and silica-filler NR compounds. All measurements are performed under identical conditions (sample thickness, beam intensity, sample-detector distance).
